# The effectiveness of stress management training on blood glucose control in patients with type 2 diabetes

**DOI:** 10.1186/s13098-018-0342-5

**Published:** 2018-05-08

**Authors:** Fereshteh Zamani-Alavijeh, Marzieh Araban, Hamid Reza Koohestani, Mahmood Karimy

**Affiliations:** 1 Department of Health Education and Promotion, School of Health, Isfahan University of Medical Sciences, Isfahan, Iran; 2 Social Determinants of Health Research Center, Ahvaz Jundishapur University of Medical Sciences, Ahvaz, Iran; 3 Department of Health Education and Promotion, Public Health School, Ahvaz Jundishapur University of Medical Sciences, Ahvaz, Iran; 4 Social Determinants of Health Research Center, Saveh University of Medical Sciences, Saveh, Iran

**Keywords:** Diabetes, Education, Social cognitive theory, Stress management, Self-efficacy

## Abstract

**Background:**

Type 2 diabetes is a chronic disease that is expanding at an alarming rate in the world. Research on individuals with type 2 diabetes showed that stressful life events cause problems in the effective management and control of diabetes. This study aimed at investigating the effect of a stress management intervention on blood glucose control in individuals with type 2 diabetes referred to Zarandeh clinic, Iran.

**Methods:**

In this experimental study, 230 individuals with type 2 diabetes (179 female and 51 male) were enrolled and assigned to experimental (n = 115) and control (n = 115) groups. A valid and reliable multi-part questionnaire including demographics, Perceived Stress Scale, Coping Inventory for Stressful Situations, Coping Self-Efficacy Scale, and multidimensional scale of perceived social support was used to for data collection. The experimental group received a training program, developed based on the social cognitive theory and with an emphasis on improving self-efficacy and perceived social support, during eight sessions of one and a half hours. Control group received only standard care. Data were analyzed using SPSS 15 applying the *t* test, paired t-tests, Pearson correlation coefficient, and Chi square analysis. The significance level was considered at 0.05.

**Results:**

Before the intervention, the mean perceived stress scores of the experimental and control groups were 33.9 ± 4.6 and 35 ± 6.5, respectively, and no significant difference was observed (p > 0.05). However, after the intervention, the mean perceived stress score of the experimental group (26.7 ± 4.7) was significantly less than that of the control group (34.5 ± 7) (p = 0.001). Before the intervention, the mean scores of HbA1c in the experimental and control groups were 8.52 ± 1 and 8.42 ± 1.2, respectively, and there was no significant difference between the two groups. However, after the intervention, the results showed a significant decrease in glycosylated hemoglobin levels in the experimental group (p ≤ 0.05). Moreover, after the intervention, the result showed a significant difference between the mean scores of all aspects of Coping Inventory for Stressful Situations, coping self-efficacy, and perceived social support in the two groups (p < 0.05).

**Conclusion:**

Our results suggested that the theory-based stress management intervention based on social cognitive theory may help to decrease stress and increase coping self-efficacy, stress management, perceived social support, and lead to a reduction in the glycosylated hemoglobin levels among patients with diabetes.

## Background

Type 2 diabetes is a chronic disease that is expanding at an alarming rate in the world. The International Diabetes Federation reports that the prevalence of diabetes has reached a global epidemic level [1].

Today, more than 415 million people in the world suffer from diabetes, which is projected to hit 642 million by 2040 [2]. The highest prevalence of diabetes has been observed in the Middle East and North Africa Region (MENA region), where Iran is located. According to the latest estimates, 35.4 million people (9.1% of adults) in this region have diabetes, which is predicted to double by the year 2030. Surprisingly, Iran is ranked third in the MENA region, in terms of the total number of adults with diabetes [2]. WHO has reported the prevalence of diabetes among Iranian men and women as 9.8 and 11.1%, respectively [3, 4]. According to WHO, currently about 3 million Iranians have diabetes and if effective action is not taken, this figure will reach 7 million by 2030. This disease is the most common cause of amputation, blindness, and chronic renal failure and it is a risk factor for developing heart disease [5].

In addition, the financial burden of diabetes has a wide range. In Canada, it was around $ 2.3 billion in 2010 and it is expected to reach $ 4.7 billion by 2020. Hence, diabetes is also an economic problem. In the US, the total cost of diabetes was around $ 174 billion in 2007, of which direct (medical) and indirect costs accounted for $ 116 billion and $ 58 billion, respectively [6, 7]. The direct costs include doctor visits, paraclinical tests, medication, surgery, hospital stays, etc. while the indirect costs include reduced productivity, disability and early death [1].

Stress is one of the main problems among patients with diabetes. Several studies have shown that stress and psychological distress play an important role in the development, intensification, and chronicity of diabetes [8]. Stress plays a dual role (cause and effect) in its relationship with diabetes. In other words, stress can be considered as a cause and yet a consequence of diabetes [9]. On one hand, stress increases glucose and glycosylated hemoglobin (HbA1C) and, on the other hand, diabetes and its consequences can increase stress levels among individuals with type 2 diabetes as well as causing other physical, behavioral, and mental disorders [10, 11]. The results of Eren’s research showed that people with diabetes experience higher levels of stress and anxiety, compared to healthy people [10]. According to the study conducted by Bradley on diabetic patients, stressful events in life cause problems in the effective management and control of diabetes [11]. Moreover, stress has been reported to elevate blood glucose levels. The hypothalamic pituitary adrenal (HPA) axis, as the central stress response system in humans, governs the neuroendocrine adaptation component of the stress response via a straightforward function. The hypothalamic release of corticotropin-releasing factor (CRF) is the main representation of this response. After binding to CRF receptors on the anterior pituitary gland, CRF (also known as CRH or corticotropin-releasing hormone) releases adrenocorticotropic hormone (ACTH). This hormone, in turn, binds to receptors on the adrenal cortex and leads to cortisol release. Cortisol is released in response to stress and stimulates the formation of glucose and glycogenolysis, leading to high blood glucose levels [12].

Because feminization with adaptive measures in coping with the stress associated with chronic diseases such as diabetes is not an easy task, patients face many problems associated with management of this disease; as it affects all aspects of personal life, including nutrition, exercise, occupation, recreation and family, and social life [13]. Studies show that about 20–40% of outpatient individuals with type 2 diabetes experience some degrees of stress and depression. Low levels of emotional and psychological health lead these patients to other problems such as increased likelihood of complications and increased mortality rates [14]. Braun et al. [15] argue that all patients with diabetes should participate in structured training and follow-up programs to improve their metabolic control and quality of life. The positive effects of blood glucose control have been proven in promoting the health of individuals with type 2 diabetes; therefore, some interventions should be designed to achieve this goal. Various theories and models have been proposed in the area of behavior change and health promotion planning. The social cognitive theory is among these theories. This model has some self-efficacy constructs for performing an activity and perceived social support [16]. Today, self-efficacy has a high status in different aspects of life and health and plays an important role in dealing with mental health problems as well as in stress and depression coping decisions. A strong self-efficacy leads to relaxation and can be a good predictor of mental health [17]. In social psychology, social support is recognized as a facilitator of healthy behavior. The effective role of social support has been proven in health, social, and psychological adaptation and in the reduction of stress and depression in chronic diseases [18]. Gao et al. showed that social support and self-efficacy in individuals with type 2 diabetes are correlated with adherence to self-care behaviors and as well as with adaptation to diabetes [18].

The present study was conducted to investigate the effect of the social cognitive theory-based intervention on blood glucose control in individuals with type 2 diabetes. In this regard, both self-efficacy and social support are effective in stress management and reduction. According, the social cognitive theory was used in this work since it covers both these constructs.

## Methodology

### Design and sample

The population of the current experimental study included all the diabetic patients admitted to Zarandieh County Diabetes Clinic (N = 420, 105 males and 315 females).

The inclusion criteria included having high-stress scores (≥ 28) based on the perceived stress scale, not having symptoms of severe psychiatric disorders, lack of late-stage complications of diabetes such as cardiovascular diseases, foot ulcers, eye problems, no history of diabetic coma, hemoglobin A1c levels above 7%, and signing informed consent form to participate in the study. On the other hand, the exclusion criteria were symptoms of severe psychiatric disorders, taking the psychiatric drug, and missing more than two sessions in the educational classroom. Based on these criteria, 187 individuals were excluded and 3 individuals declined to participate, and finally 230 individuals (179 female and 51 male) enrolled in the study. They were randomly assigned to two experimental (n = 115) and control (n = 115) groups.

### Procedure

Randomization was achieved using sealed numbered envelopes developed from a random number generator. A research assistant who was not involved in the recruitment of participants prepared the envelopes. Participants allocated to the control group (n = 115) received standard care. Participants assigned to the intervention group (n = 115) also received standard care plus the stress management intervention. Three months after the intervention, the posttest was conducted for both experimental and control groups to examine the effects of education on the primary and secondary outcomes. It is noteworthy that standard care in Iran includes weekly visits at a healthcare clinic from a doctor, a dietitian, and a nurse and lasts for less than 20 min. These visits were held individually or in groups. Because of the nature of the intervention in the current study, the instructor was not blinded to group assignment, but participants and statistical investigator were blinded to group assignment.

### Research tools

The primary outcomes of the current research include the Perceived Stress Scale, Endler and Parker’s Coping Inventory for Stressful Situations, the Coping Self-Efficacy Scale, and the Multidimensional Scale of Perceived Social Support. The secondary outcome was HbA1c levels. HbA1c tests were conducted using a biosystem kit and chromatography method. Biosystem kits are standard kits approved by the Iranian Ministry of Health and Medical Education. The kit was used two times; before the beginning of the study and 3 months after the end of the intervention.

The following questionnaires were also used:

*(1) The demographic questionnaire* This questionnaire measures variables including age, gender, educational qualifications, duration of diabetes, body mass index (BMI), and marital status. BMI ranged 18.5–24.9 was considered as normal; under 18.5 as underweight, over 25 as overweight and over 30 as obese [6].

*(2) The Perceived Stress Scale* The Cohen’s Perceived Stress Scale was used to measure perceived stress within the past month. It uses 14 items to measure thoughts and feelings about the events to control, overcome and to cope with the past mental stresses and distresses. It also examines the risk factors associated with behavioral disorders and reveals the process of stressful relationships. The scale uses a 5-point Likert scale and answers range from never (score 0), rarely (score 1), sometimes (score 2), often (score 3), and most times (score 4). The lowest possible score is 0 and the highest score is 56 [19]. Higher scores indicate higher levels of perceived stress. This questionnaire has been validated by Sigari et al. in Iran and its internal consistency has been confirmed (Cronbach’s alpha coefficient = 0.90) [19]. In the present study, the internal consistency of the questionnaire was 0.88.

*(3) Endler and Parker’s Coping Inventory for Stressful Situations (CISS):* This scale contains 48 items and includes *task*-*oriented* coping strategies (16 items), *emotion*-*oriented* coping strategies (16 items), and a*voidance*-*oriented* coping strategies (16 items). The questionnaire uses a 5-point Likert scale and answers range from never (score 1) to always (score 5). The participants’ scores determine which coping strategy better suits them. The lowest and the highest possible scores include 16 and 80 for each strategy [19]. The internal consistency of the Persian version of the questionnaire has been confirmed (Cronbach’s alpha coefficient = 0.83) [20]. In the present study, the Cronbach’s alpha coefficients of 0.85, 0.86, 0.83, and 0.82 were obtained for the whole scale, the problem-focused, the emotion-focused, and avoidance coping strategies, respectively.

*(4) The Coping Self*-*Efficacy (CSE) Scale* The CSE of Chesney et al. is a 26-item test, which is designed on an 11-point Likert scale ranged from (I can never cope with it (score 0) to I am sure that I can cope with it (score 10). This scale has three subscales; using problem-solving coping strategies (12 items), stopping negative thoughts and emotions (9 items), and gaining friends and family support (5 items). To calculate the total score of each sub-scale, the scores of all the items associated with the sub-scale must be added. Thus, the subscales of problem-solving coping strategies, stopping negative thoughts and emotions, and gaining friends and family support are ranged from 0 to 120, 0 to 90, and 0 to 50, respectively [21]. Mahmoudi et al. measured the internal consistency of the above subscales for the Persian version of the questionnaire and obtained these coefficients: 0.84, 0.83, and 0.73, respectively [21]. In the present study, the Cronbach’s alpha coefficients of the subscales included 0.90, 0.88, and 0.85, respectively.

The total score of each sub-scale is calculated by adding the scores of all the items associated with the sub-scale.

*(5) The multidimensional scale of perceived social support (MSPSS)* This scale contains 12 questions and measures three factors, including friends’ support, family, and significant others. This scale uses a 7-point Likert scale and has an answer range from 0 to 6 (I strongly disagree to I strongly agree). The scores range from 0 to 72. This scale has been validated by Bagherian-Sararoudi et al. [22] in Iran and its internal consistency has been confirmed (Cronbach’s alpha coefficient = 0.84). In the present study, the internal consistency of the questionnaire was 0.86.

### Intervention

The training sessions were performed in Zarandieh County Diabetes Clinic. The intervention program was developed based on the social cognitive theory and had an emphasis on improving self-efficacy and perceived social support. Participants in the experimental groups received eight sessions of one and a half hours educational sessions in groups of 6–10 persons. According to SCT, the behavior is being learned within the context of social interactions, experiences, and media influence. Thus, people learn new behaviors by both trying them and subsequent outcome of the behaviors and also by observing the replication of the behavior by a third party or a role model. To increase relaxation self-efficacy, we focused on the antecedents (e.g., sources of information) that may be used to influence self-efficacy related to stress management and relaxation, performance accomplishment (e.g., past experience with relaxation and aggressiveness control), vicarious experience (e.g., observation of role models’ relaxation technique practices), verbal persuasion (e.g., encouragement and support from others), and physiologic cues (e.g., anxiety and fatigue). Examples of the strategies used included providing information, discussion, and practice with a psychologist to enhance performance accomplishment in regard to relaxation; having a patient with diabetes attend the education session as a role model to demonstrate relaxation skills and answer questions (vicarious experience); giving positive verbal feedback (verbal persuasion); and providing anticipatory guidance to acknowledge and normalize relaxation challenges (decrease anxiety). To better influence supports from others, two educational sessions were held for participants spouses and significant others mentioned by them in the questionnaire to help patients with using relaxation techniques. Some training pamphlets and booklets were also provided for each patient. The information booklet, prepared by a health education specialist and validated by the expert panel, included written text (brief messages about diabetes self-care and relaxation) with illustrations to reinforce the information. The images of damaged human’s body organs were accompanied by messages such as “by doing relaxation practices you reduce the risk of developing the end-stage disease and chronic conditions”. A phone number had been written at the end of the booklet for participants who may seek more information.

The following eight group sessions were held with the help of a psychologist during a 1-month period (twice a week); session one: teaching muscle relaxation techniques as reported earlier [23], communication skills, and getting acquainted with external supportive agents; session two: social communication skills such as empathy; session three: examining the three factors effective in self-efficacy (thought, affection and action) and examining rational and irrational thinking; session four: providing strategies to cope with irrational thoughts and to replace them rational thoughts; session five: providing strategies to cope with irrational thoughts and to replace them rational thoughts; session six: teaching three coping strategies (aggressiveness, passiveness and assertiveness) and providing assertive strategies; session seven: introducing ineffective and effective strategies for coping with stressful situations (inefficient avoidance strategies, etc.) and finally session eight: providing strategies for coping with stress (problem-focused and emotion-focused coping strategies). The skills were then practiced using written scenarios.

### Data analysis

The results were analyzed in SPSS 15 using paired and independent t-tests, Pearson correlation coefficient, and Chi square analysis. The significance level was considered at 0.05. The Kolmogorov–Smirnov test was used to check the normality of data distribution.

### Ethical considerations

This project is approved by the Ethics Committee of Saveh University of Medical Sciences (IR.SAVEHUMS.REC.1396.17). After granting the consent of the patients, the aim of the study, the methodology, and the advantages and disadvantages of the study were explained to them. The participants were assured that they are not obliged to participate in the research and that they can leave it whenever they wish. They were also assured of the confidentiality of their information.

## Results

**Fig. 1:**
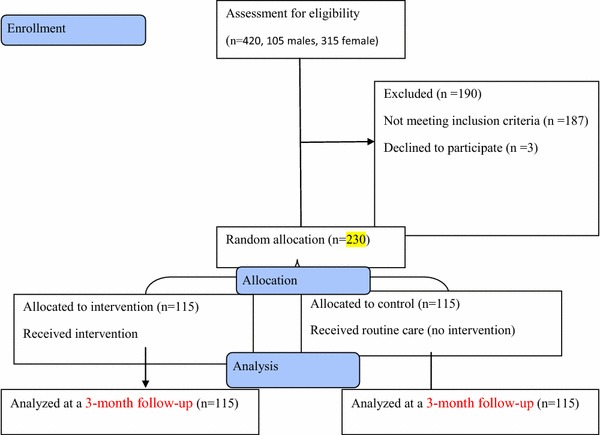
Flow diagram of the participants

In total, 230 (179 female and 51 male) patients with type 2 diabetes were enrolled. Figure 1 shows a flow diagram of the participants during the study period.

The mean ages of patients in the experimental and control groups were 56.4 ± 7.2 and 57.1 ± 6.5 years, respectively. The demographic characteristics of the participants are presented in Table 1. There was no statistically significant difference between the two groups in terms of age, gender, duration of diabetes, marital status, BMI, and educational qualifications.

**Table 1:** The frequency distribution of the patients in the two groups based on the qualitative demographic variables

Group variable	Experimental group		Control group		P-value*
	No.	%	No.	%	
Gender					
Male	23	20	28	24	0.526
Female	92	80	87	76	
Marital status					
Single	18	15.6	14	12.2	0.568
Married	97	84.4	101	87.8	
Educational qualifications					
Illiterate	6	5	5	4	0.849
Elementary and middle school	62	54	59	51	
Diploma and higher degrees	47	41	51	44	
Duration of diabetes (years)					
< 2	15	13	21	18	0.547
2–4	51	44	47	41	
> 4	49	42	47	41	
Age (years)					
< 30	8	7	9	8	0.947
31–49	48	42	46	40	
> 50	59	51	60	52	
BMI					
Underweight	5	4	6	5	0.956
Normal	22	19	21	18	
Overweight	52	45	49	43	
Obese	36	32	39	34	

Derived from Chi square

Based on the results, before the intervention, the mean perceived stress scores of the experimental and control groups were 33.9 ± 4.6 and 35 ± 6.5, respectively, with no statistically significant difference observed (p = 0.38). However, after the intervention, the mean perceived stress score of the experimental group (26.7 ± 4.7) was significantly less than that of the control group (34.5 ± 7) (p = 0.001). Before the intervention, the patients in the two groups were homogeneous in terms of various aspects of Coping Inventory for Stressful Situations (CISS) (*task*-*oriented* coping, *emotion*-*oriented* coping, and *avoidance*-*oriented* coping), various aspects of Coping Self-Efficacy (problem-solving coping strategies, stopping negative thoughts and emotions, and gaining friends and family support) and perceived social support. However, after the intervention, the independent t-test showed a significant difference between the mean scores of all aspects of Coping Inventory for Stressful Situations (CISS), coping self-efficacy, and perceived social support in the two groups (p < 0.05) (Table 2).

**Table 2:** Comparison of constructs in two groups at baseline and 3-months follow-up

Variable	Time group	Baseline mean ± SD	3-months follow-up mean ± SD	P-value*
Stress	Experimental group	4.6 ± 33.9	4.7 ± 26.7	0.001
	Control group	6.5 ± 35	7 ± 34.5	0.14
	P-value**	0.38	0.001	–
Problem-Focused Coping Strategies	Experimental group	9 ± 43.6	3.9 ± 51.4	0.001
	Control group	5.9 ± 46.1	8.1 ± 45.7	0.16
	P-value**	0.13	0.001	–
Emotion-Focused Coping Strategies	Experimental group	5.5 ± 35.6	4.9 ± 42.9	0.001
	Control group	8.2 ± 37.8	8.1 ± 38.8	0.544
	P-value**	0.17	0.009	–
Avoidance-focused coping strategies	Experimental group	41.0 ± 5.1	2.7 ± 45.3	0.001
	Control group	5.9 ± 42.4	4.9 ± 41.6	0.19
	P-value**	0.29	0.001	–
Self-efficacy use problem-focused coping	Experimental group	10.5 ± 57.2	11.4 ± 67.3	0.001
	Control group	11.6 ± 61.8	11.2 ± 60.7	0.233
	P-value**	0.07	0.02	–
Self-efficacy stop unpleasant emotions and thoughts	Experimental group	9.2 ± 51.3	10.5 ± 59.5	0.001
	Control group	11.2 ± 52.1	11.2 ± 53.2	0.234
	P-value**	0.71	0.03	
Self-efficacy gaining friends and family support	Experimental group	8.1 ± 28.5	34.5 ± 5.9	0.001
	Control group	9 ± 31.9	30.2 ± 9.7	0.08
	P-value**	0.08	0.03	
Social support	Experimental group	8.1 ± 38.5	6.3 ± 46.7	0.401
	Control group	7.4 ± 39.4	8.4 ± 40.3	0.007
	P-value**	0.63	0.004	–

* Paired T-test; ** Independent T-test

As shown in Table 3, before the intervention, the mean scores of HbA1c in the experimental and control groups were 8.52 ± 1 and 8.42 ± 1.2, respectively, and the independent t-test showed no statistically significant difference between the two groups. However, after the intervention, the results showed a significant reduction in glycosylated hemoglobin levels in the experimental group (p ≤ 0.05). In addition, the paired t-test showed a significant difference between the mean scores of HbA1c in the experimental group before and after the intervention while the difference was not statistically significant in the control group.

**Table 3:** Comparison of glycosylated hemoglobin (HbA1c) levels in two groups at baseline and 3-months follow-up

Variable	Time group	Baseline mean ± SD	3-months follow-up mean ± SD	P-value*
HbA1c	Experimental group	8.52 ± 1	6.1 ± 1	0.001
	Control group	8.42 ± 1.2	8.21 ± 1.31	0.530
	P-value**	0.706	0.0001	

## Discussion

In addition to the physical complications, diabetes-induced stress has some adverse psychiatric complications that make diabetes difficult to control in terms of identification and elimination processes [24]. In chronic diseases such as diabetes, psychotherapy can reduce the need for expensive medical services and increase the mental health of patients [25]; thus, this study focuses on Coping Inventory for Stressful Situations (CISS) and used self-efficacy and social support constructs to improve the condition of these patients.

The results showed that stress management techniques could help patients to control their blood glucose levels, which in turn can prevent long-term complications of diabetes, such as diabetic foot ulcer and blindness. Stratton believes that a 5% reduction in HbA1c levels is associated with a significant reduction in the risk of coronary artery disease. In line with the present study, Hamid’s study in Iran showed that stress management training was effective in controlling blood glucose and decreasing depression, anxiety, stress, and HbA1c levels in diabetic women [26]. The study of Attari et al. also demonstrated the beneficial effects of stress management program in controlling blood glucose levels in diabetic patients [27]. In another study conducted by Surwit et al. at Duke University Outpatient Clinics, the results showed that stress management training program is a useful and cost-effective technique in reducing the HbA1c levels in diabetic patients [28]. The results of the present study, in line with the previous studies, highlight the importance of applying stress management techniques in controlling blood glucose levels in diabetic patients. Therefore, it seems that employing psychologists in diabetes clinics or holding retraining management courses for medical staff can improve the health of diabetic patients.

According to the cognitive theory, there is a strong negative correlation between self-efficacy and stress and the present research showed that people with higher levels of self-efficacy might better manage their stress. The present study also confirmed the negative correlation between self-efficacy and perceived stress [29]. After the intervention, the self-efficacy levels increased while the perceived stress and HbA1c levels decreased significantly. Schwerdtfeger et al. found that high self-efficacy might act as a supportive factor for psychological well-being by strengthening the immune system, reducing the release of stress-related hormones, and improving the mental health of people [30]. In line with our results, Gao et al. found that higher levels of self-efficacy was associated with better self-care and decreased HbA1c levels in diabetic patients [18]. Similarly, Walker et al. in their study in the US found that higher levels of self-efficacy were associated with better self-care, better blood glucose control, and a higher quality of life of diabetic patients [31]. Al-Khawaldeh et al. in their study in Jordan found that higher levels of self-efficacy were associated with a daily walk, diet and drug control, and better control of the blood glucose levels in diabetic patients [32]. Schoenthaler et al. studied patients with chronic diseases and observed that patients with higher levels of self-efficacy better perform recommended health behaviors than those with lower levels [33].

Previous studies have shown that social support plays an important role in the self-care of patients with chronic diseases [34, 35]. For example, a meta-analysis of 122 studies conducted by DiMatteo showed that adherence to medical regimens in patients with social support increases by 27% [36]. In line with previous studies, our results also showed that increasing perceived social support would decrease perceived stress levels in patients, which consequently results in a reduction in the HbA1c levels. Miller’s study proved that social support can increase levels of self-esteem and self-efficacy as well as reducing levels of stress and depression among patients [37]. In the studies of Aikens et al. [38] and Pereira et al. [39], social support had the greatest correlation with blood glucose control. Glasgow and Toobert in their study showed that family support was the most important factor in adherence to strict diets as well as in blood glucose control [40]; however, the study of Chew et al. in Malaysia did not show any significant relationship between social support and blood glucose control. This contradictory conclusion may be due to the different tools used in Chew’s study and because of discarding the variable of friends and family support [34]. A review study by Stopford et al. on 29 studies revealed that among the components of social support, family support is effective in reducing HbA1c levels and in blood glucose control [35].

### Limitation

Since the current research was performed in one geographical area in Iran, the generalizability of our results is decreased. Also, we followed up the patients for 3 months as the longer follow up may lead to more accurate outcomes. In addition, other factors that can affect the results such as diet and physical activity were not measured in this study, and thus could be addressed in future studies.

## Conclusion

Our results suggested that the theory-based stress management intervention based on social cognitive theory may help to decrease stress and coping self-efficacy, stress management, and perceived social support and lead to a reduction of the glycosylated hemoglobin levels among patients with diabetes in Zarandieh, Iran.

## Abbreviations

CISS: Coping Inventory For Stressful Situations
CSE: Coping Self-Efficacy
BMI: body mass index
CVR: content validity ratio
CVI: content validity index
